# Blood leukocyte count as a systemic inflammatory biomarker associated with a more rapid spirometric decline in a large cohort of iron and steel industry workers

**DOI:** 10.1186/s12931-021-01849-y

**Published:** 2021-09-26

**Authors:** Nan Kong, Guoshun Chen, Haitao Wang, Jianyu Li, Shuzhen Yin, Xue Cao, Tao Wang, Xin Li, Yanan Li, Huanling Zhang, Shanfa Yu, Jinglong Tang, Akshay Sood, Yuxin Zheng, Shuguang Leng

**Affiliations:** 1 Department of Occupational and Environmental Health, School of Public Health, Qingdao University, Qingdao, 266021 Shandong China; 2 Wugang Institute for Occupational Health, Wuyang Iron and Steel Company Limited of Hangang Group in Henan, Wuyang, Henan China; 3 Henan Medical College, Zhengzhou, Henan China; 4 Department of Internal Medicine, School of Medicine, University of New Mexico, Albuquerque, NM 87131 USA; 5 Cancer Control and Population Sciences, University of New Mexico Comprehensive Cancer Center, Albuquerque, NM USA

**Keywords:** Longitudinal study, Steel dust exposure, White blood cell count, Lung function decline, Systemic inflammation

## Abstract

**Objective:**

Iron and steel industry workers are exposed to high levels of inhalable dust particles that contain various elements, including metals, and cause occupational lung diseases. We aim to assess the relationship between occupational dust exposure, systemic inflammation, and spirometric decline in a cohort of Chinese iron and steel workers.

**Methods:**

We studied 7513 workers who participated in a Health Surveillance program at Wugang Institute for Occupational Health between 2008 and 2017. Time-weighted exposure intensity (TWEI) of dust was quantified based on self-reported dust exposure history, the experience of occupational hygienists, and historical data of dust exposure for workers with certain job titles. A linear mixed-effects model was used for association analyses.

**Results:**

The average annual change of lung function was − 50.78 ml/year in forced expiratory volume in 1 s (FEV1) and − 34.36 ml/year in forced vital capacity (FVC) in males, and − 39.06 ml/year in FEV1 and − 26.66 ml/year in FVC in females. Higher TWEI prior to baseline was associated with lower longitudinal measurements of FEV1 and FVC but not with their decline rates. Higher WBC and its differential at baseline were associated with lower longitudinal measurements and a more rapid decline of FEV1 and FVC in a dose-dependent monotonically increasing manner. Moreover, the increase of WBC and its differential post-baseline was also associated with a more rapid decline of FEV1 and FVC.

**Conclusions:**

Our findings support the important role of systemic inflammation in affecting the temporal change of lung function in iron and steel industry workers.

**Supplementary Information:**

The online version contains supplementary material available at 10.1186/s12931-021-01849-y.

## Introduction

The iron and steel industry as a fundamental component of modern industrial infrastructure for the entire human society has employed millions of workers who were exposed to many chemical and physical hazards, workplace activities, or conditions [1]. Workers are exposed to high levels of inhalable dust particles containing various elements such as metals, silica, carbon, and polycyclic aromatic hydrocarbon, which could cause chronic occupational diseases such as chronic obstructive pulmonary disease (COPD) [2–5]. The International Agency for Research on Cancer has classified iron and steel founding processes as Group 1 human carcinogens based on sufficient evidence for lung cancer in humans [6]. Cross-sectional studies have established strong associations between dust exposure and lung function impairment in iron and steel workers [1, 7, 8]. However, the association between dust exposure in the working environment and lung function decline is inadequately studied.

Inhalation of dust particles containing metals and their compounds causes impairment to both pulmonary surfactant and respiratory function [4]. Occupational exposure to respirable dust in an iron foundry played a significant role in decreasing lung function and in increasing the risk of chronic airway obstruction in exposed workers [1, 2, 9]. In a longitudinal study from the Norwegian, the association of steel dust exposure with accelerated lung function decline was observed and smokers had stronger associations. A dose–response relationship between total dust exposure and the annual decline in FEV1 has been found among employees in smelters producing ferromanganese, silicomanganese, ferrochromium and silicon carbide [10].

White blood cell (WBC) counts and its differential (i.e., neutrophils, lymphocytes, monocytes, eosinophils, and basophils) are established systemic inflammatory markers and have been identified to be associated with lower FEV1 and airflow obstruction in occupational cohorts and general populations [11–16]. After the September 11, 2001 World Trade Center attacks, rescue and recovery workers were exposed to a high level of dust mixture and were later found to have high rates of airway injury, including excessive loss of lung function, airflow obstruction, and airway hyper-reactivity. Elevated blood neutrophil and eosinophil counts were independently associated with an accelerated FEV1 decline (64 mL/year or more), a well-established risk factor for COPD development [17]. Mechanisms underlying the observed associations may involve increased lung tissue damage due to the release of destructive enzymes or highly reactive oxygen species from neutrophils [18–21] or the generation of eosinophilia/Th2 inflammation in airways [14, 22].

Few studies have evaluated the associations between WBC counts and longitudinal changes of lung function in iron and steel workers. We hypothesized baseline WBC as a systemic inflammatory biomarker is associated with a lower lung function and a more rapid decline in a cohort of 7513 iron and steel industry workers. We also assessed the increase of WBC counts post-baseline and its association with the longitudinal change of spirometry.

## Methods

A detailed description is available in supplemental materials.

### Study subjects

This longitudinal study was conducted in 7575 employees from Wuyang Iron and Steel Company Limited (Hangang Group in Henan, China). The employees were required to participate in the Worker Health Surveillance program at the Wugang Institute for Occupational Health between 2008 and 2017. This company has been using the electric arc furnace technique to manufacture steel from scrap or direct reduced iron, melted by electric arcs, and mainly has steel making, continuous casting, rolling, and oxygen-making plants. According to the Reports of Occupational Hazard Control Assessment conducted by Henan Institute of Occupational Medicine in 2006 and 2007, inhalable dust and noise are the two occupational hazards that have samples exceeding the national standards (the permissible concentration time-weighted average of 8 mg/m^3^ for inhalable dust and 85 dB(A) for noise in China). This cohort was dynamic with workers entering and leaving the cohort at different times. In general, individual participants in this program received medical assessments every other year with medical surveillance workouts recommended by the China Ministry of Health. The de-identified data were obtained from the Wugang Institute for Occupational Health. The Research Ethics Committee of the Qingdao University School of Medicine approved the study protocol with a waiver of subject consent (QYFYWZLL25933).

### Occupational dust exposure assessment

Employment history including occupation, length of employment, and occupational dust exposure (yes or no) for three consecutive jobs or positions were self-reported at study entry. Because no cohort members were newly employed at the baseline visit, we focused on detailed employment history including plant, workshop, and post at the Wuyang Iron and Steel Company prior to baseline visit to quantify occupational dust exposure. All study subjects belonged to 79 workshops from seven plants and the population was divided into three groups by tertiles based on the percentage of subjects reporting dust exposure. Combined with the consultation of occupational hygienists, historical personal air sampling data in 2006 and 2007 (Additional file 1: Table S1), and epidemiological consideration of sample size within each exposure category, the workshops were classified into low (n = 43, person-posts = 4034), medium (n = 20, person-posts = 3816), and high (n = 16, person-posts = 2562) exposure categories to maximize the statistical power of the study. We calculated time-weighted exposure intensity (TWEI) using the sum of exposure unit (coded as 0, 1, 2 for low, medium, and high) years divided by total years prior to the baseline of dust exposure for each individual.

### Lung function paraments

Spirometry was performed for each individual without a bronchodilator every other year by a certified respiratory technician using a portable calibrated electronic spirometer (CHESTGRAPH HI-701, Japan) in accordance with the American Thoracic Society (ATS)/European Respiratory Society standards(ERS) [23]. The spirometer was checked every day for leaks by a calibrated syringe. Persons were in the standing position with a nose clip used. After two or more practice blows, FEV1 and FVC were determined as the highest value from the results of measurements. Standing height and body weight were measured and recorded at each test occasion. Forced expiratory volume in 1 s (FEV1), forced vital capacity (FVC), and FEV1/FVC ratio were used in this study. Percent predicted values were calculated using the equations for Asian adults supplied in the user’s manual.

### Complete blood count with differential

Peripheral venous blood samples were drawn from the antecubital veins of patients after overnight fasting. The blood samples were collected into lithium heparin-containing tubes to avoid pseudo thrombocytopenia. The number of red blood cells (including red blood cells, hemoglobin and hematocrit, etc.), white blood cells (WBC), platelets, and WBC differential counts (i.e., neutrophils, lymphocytes, monocytes, basophils, and eosinophils) were measured by a hematology analyzer (Sysmex. XS-500ix, China). Mid-range absolute counts (MID) include monocytes, eosinophils, basophils, blasts and other precursor white cells that fall in a particular size range. Absolute cell counts were used in the analyses. Baseline and follow-up blood samples were analyzed using the same machine operated by trained technicians.

### Data analysis

**Fig. 1:**
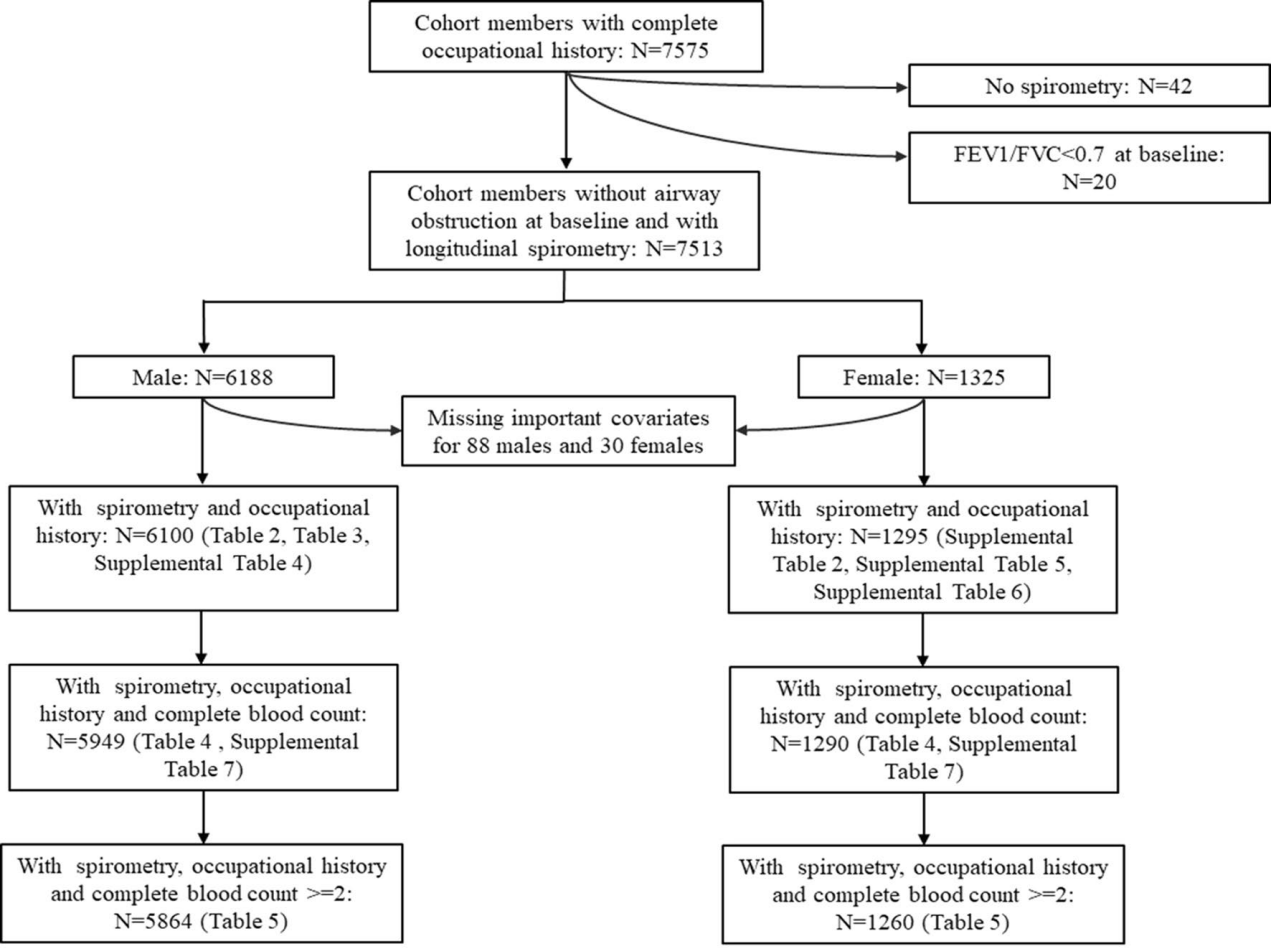
Study design and sample size evolution

Workers with airway obstruction (defined as FEV1/FVC < 0.7) at baseline (n = 20) or without spirometry data (n = 42) were excluded from this study, thus leaving 7513 workers with at least one spirometry measurement for data analyses. The sample size evolution for different analyses was summarized in Fig. 1. Females were more likely to conduct less physically intensive assignments, thus had less exposure to occupational hazards (Table 1). Therefore, all data analyses were conducted in males and females separately because of the division of labor by sex in heavy industry. A prudent analytical plan was developed to analyze the relationship among dust exposure, WBC count, and longitudinal spirometric decline with a careful assessment of important covariates, dose–response relationship, and potential confounding effects by linear mixed-effects model. First, a linear mixed-effects model with a subject-specific random intercept was used to assess the associations between occupational dust exposure (i.e., TWEI and years of dust exposure) prior to baseline and longitudinal spirometry with adjustment of important covariates. An interaction term between time-in-cohort (TIC) and prior occupational dust exposure was included in the model to assess whether lung function decline varied by previous occupational dust exposure. Baseline spirometry and its interaction with TIC were included in the models to minimize its effect on the association between occupational dust exposure and lung function decline. Second, similar approaches were taken to assess the association of prior occupational dust exposure on longitudinal measurements of WBC count and differential. Third, we also used linear mixed-effects models to assess the association of WBC count and differential at baseline with longitudinal measurements of lung function and its decline. WBC count and differential were included in the model as continuous variables first, then converted into categorical variables to assess their dose–response relationship. Using WBC count and differential collected at the same visits of spirometry in the model did not change the associations observed in models using baseline data for WBC count and differential. We further analyzed the elevation of WBC and its differential post-baseline and their association with lung function decline. Finally, three-way interactions (e.g., current smoker × TWEI × TIC or current smoker × WBC × TIC) with their two-way interactions and main effects were included in the models to analyze the potential effect modification of cigarette smoking on the associations of longitudinal lung function with occupational dust exposure or WBC count and differential. Data analyses were performed using SAS version 9.4 (site 70239492).

**Table 1:** Characteristics of study subjects by sex

Variable	Male			Female			P ^a^
	n	M ± SD	Median (Q1, Q3)	n	M ± SD	Median (Q1, Q3)	
Age (year)	6188	34.4 ± 9.2	34 (26, 42)	1325	34.7 ± 7.6	35 (29, 40)	0.014
Han Ethnic (n, %)	6188	6129, 99.1		1325	1312, 99.0		0.972
Height (cm)	6110	171.5 ± 5.5	171 (168, 175)	1295	160.1 ± 5.2	160 (156, 164)	< 0.001
BMI (kg/m^2^)	6110	24.5 ± 3.6	24.38 (21.9, 26.9)	1295	22.3 ± 3.1	21.87 (20.03, 23.88)	< 0.001
Current smoker (n, %)	6188	3034, 49.0		1325	0, 0		NC
Packyears	3034	11.0 ± 10.4	7.5 (3.0, 18.0)	0	NA	NA	NC
Years of dust exposure (year)	6188	12.6 ± 9.7	10.5 (3.3, 20.8)	1325	13.5 ± 8.2	13.9 (5.3, 19.9)	< 0.001
Time in cohort (year)	6188	5.4 ± 2.8	6.00 (3.9, 8.1)	1325	5.3 ± 2.6	6.0 (3.9, 7.9)	0.184
TWEI	6188	0.93 ± 0.74	1.00 (0.00, 1.5)	1325	0.64 ± 0.74	0.19 (0.00, 1.00)	< 0.001
NO. of spirometry (n)	6188	3.5 ± 1.4	4 (2, 5)	1325	3.4 ± 1.2	3 (2, 4)	0.004
Spirometry							
FEV1 (ml/s)	6188	3838.6 ± 622.5	3800 (3410, 4240)	1325	2828.7 ± 440.2	2800 (2520, 3090)	< 0.001
FEV1% predicted (%)	6105	102.2 ± 14.3	100.7 (92.0, 111.0)	1295	101.5 ± 14.6	100.0 (90.9, 109.9)	0.098
FVC (ml)	6188	4211.4 ± 623.0	4150 (3730, 4650)	1325	3101.5 ± 467.6	3060 (2750, 3400)	< 0.001
FVC% predicted (%)	6105	103.1 ± 14.2	101.5 (92.5, 112.3)	1295	106.0 ± 14.7	104.4 (94.9, 115.4)	< 0.001
FEV1/FVC (%)	6188	91.3 ± 6.2	91.3 (87.0, 96.5)	1325	91.3 ± 5.9	91.2 (87.5, 95.9)	0.702
White blood cell count							
WBC (10^9^ cells per L)	5949	6.23 ± 1.63	6.0 (5.1, 7.1)	1290	5.31 ± 1.41	5.1 (4.3, 6.1)	< 0.001
NEU (10^9^ cells per L)	5945	4.00 ± 1.35	3.8 (3.1, 4.7)	1287	3.43 ± 1.13	3.3 (2.6, 4.0)	< 0.001
LYM (10^9^ cells per L)	5945	21.97 ± 0.54	1.9 (1.6, 2.3)	1287	1.67 ± 0.51	1.6 (1.4, 1.9)	< 0.001
MID (10^9^ cells per L)	5945	0.26 ± 0.14	0.2 (0.2, 0.3)	1287	0.21 ± 0.11	0.2 (0.1, 0.3)	< 0.001
HGB (g/L)	5035	149.35 ± 12.43	150 (141, 158)	1174	123.86 ± 13.16	124 (117, 133)	0.194

The prediction reference equation for Asian adults: (1) FVC (ml): (27.63–0.112*age) *height for male, (21.78–0.101*age) *height for female; (2) FEV1 (ml): 34.4*height-33*age-1000 for male, 26.7*height-27*age-540 for female

*M*  mean, *SD*  standard deviation, *Q*  quartile, *BMI*  body mass index, *TWEI*  time-weighted exposure intensity, *FEV1*  forced expiratory volume in 1 s, *FVC*  forced vital capacity, *WBC*  white blood count, *NEU*  neutrophilicgranulocyte, *LYM*  lymphocyte, *MID*  mid-range absolute count

^a^T-test for all variables between sexes except for ethnicity which used χ^2^ Test

## Results

### Demographics of study subjects

This study included 6188 male and 1325 female workers with an average age of 34.5 years at study entry (Table 1). On average, workers reported 13 years of occupational dust exposure at the Wuyang Iron and Steel Company, concordant with the fact that the Company was the first employer for most cohort members. The median follow-up duration in the cohort (TIC) was 6 years, which allowed for three to four spirometry measurements. In total, 25,164 spirometry measurements were obtained from 7513 workers. The average percent predicted values of FEV1 and FVC at baseline were over 100%. Airflow obstruction was identified in 22 subjects during follow-up evaluations, resulting in an incidence of 2.9 cases per 1000 person-years.

### Occupational dust exposure

Median inhalable dust concentrations (8-h time-weighted average [TWA]) for workshops classified in medium and high exposure categories were 0.91–1.45 and 7.45–10.35 mg/m^3^, between 2006 and 2007, respectively. Ninety percent of dust mass was non-silica (potentially as metal dust). Size distribution analysis found over 80% of particles had sizes less than 5 μm. No occupational monitoring data was available for the low exposure category. However, PM_2.5_ levels ranged 33 to 108 μg/m^3^ in non-heating seasons and 73 to 203 μg/m^3^ in heating seasons reported by the national air quality monitoring stations closest to Wugang city (about 30 miles away) between 2013 and 2017. The entire company area could be polluted by industrial dust due to its location in a small valley, which is 5 km long and 1.9 km wide with north and west sides surrounded by mountains. The median TWEI was 1.0 units for male workers and 0.19 units for female workers, supporting male workers having much higher occupational dust exposure.

### Occupational dust exposure and spirometry

The average annual change of FEV1, FVC, and FEV1/FVC ratio during follow-up were 50.78 ml, 34.36 ml, and 0.49 in males (Table 2, model 1) and 39.06 ml, 26.66 ml, and 0.50 in females (Additional file 1: Table S2, model 1), respectively. Higher TWEI prior to baseline was associated with lower longitudinal measurements of FEV1 and FVC in either sexes and with females more vulnerable to the adverse effect of occupational dust exposure (e.g., − 21.76 ml in males versus − 41.96 ml in females for FEV1 and − 28.71 ml in males versus − 36.67 ml in females for FVC, per unit increase of TWEI, Table 2 and Additional file 1: Table S2, model 1), but had no effect on FEV1/FVC ratio. The most probable reason for lower spirometry in workers with higher past occupational dust exposure may be their more rapid initial decline of lung function or inadequate ongoing lung growth and development after the initiation of exposure. However, occupational dust exposure prior to baseline was associated with a slower annual decline of FEV1 and FVC in either sexes (Table 2 and Additional file 1: Table S2, model 2). We hypothesized a slower decline of FEV1 and FVC associated with higher TWEI could be due to the “healthy worker effect” and certain worker characteristics could mediate such associations. Univariate analyses identified workers with higher TWEI were younger and taller, and had shorter years of dust exposure for either sexes at baseline (Additional file 1: Table S3). Age and years of dust exposure were highly correlated with Spearman correlation coefficients > 0.93 for either sexes and could not be the factors mediating the observed associations because occupational dust exposure history and its interaction with TIC had been included in the model (Table 2 and Additional file 1: Table S2). Taller workers had a slower decline of FEV1 and FVC and a faster decline of FEV1/FVC ratio for either sexes and inclusion of interaction term of height and TIC in model 2 completely nullified the significance of interaction term of TWEI and TIC (Additional file 1: Tables S4 and S5), suggesting that height may be the healthy worker characteristic that mediates the association between higher TWEI and slower decline of spirometry. Moreover, the inclusion of any additional interaction terms with TIC (e.g., smoking status, HGB, packyears, and BMI) had no impact on the estimate and significance for the interaction term of TWEI and TIC (data not shown).

**Table 2:** The association between dust exposure and spirometry in male workers using linear mixed-effects model (n = 6100)

Variable	FEV1 (ml/s)						FVC (ml)						FEV1/FVC (%)						
	Model 1			Model 2			Model 1			Model 2			Model 1				Model 2		
	β	SE	P	β	SE	P	β	SE	P	β	SE	P	β	SE	P	β		SE	P
Intercept	− 1263	172.2	< 0.001	− 1400.4	103.1	< 0.001	− 2269.7	178.7	< 0.001	− 2100.1	114.8	< 0.001	107.63	2.2	< 0.001	17.33		1.5	< 0.001
Age (yr)	− 22.85	1.6	< 0.001	− 7.76	1.0	< 0.001	− 21.38	1.7	< 0.001	− 9.19	1.1	< 0.001	− 0.10	0.02	< 0.001	− 0.01		0.001	0.400
Current smoker	15.37	10.3	0.135	− 3.13	7.3	0.668	18.87	11.3	0.095	2.15	8.2	0.794	− 0.07	0.1	0.611	− 0.11		0.1	0.251
Packyears (py)	− 2.02	0.7	0.004	− 0.77	0.5	0.100	− 1.87	0.8	0.013	− 0.9	0.5	0.086	− 0.01	0.01	0.117	− 0.004		0.006	0.436
BMI (kg/m^2^)	− 10.52	1.5	< 0.001	− 0.62	0.9	0.473	− 10.85	1.5	< 0.001	− 2.88	1.0	0.003	− 0.02	0.01	0.233	0.03		0.01	0.002
Height (cm)	35.89	1.0	< 0.001	13.95	0.6	< 0.001	43.82	1.0	< 0.001	19.74	0.7	< 0.001	− 0.08	0.01	< 0.001	− 0.04		0.007	< 0.001
TIC (yr)	− 50.78	0.8	< 0.001	199.38	5.6	< 0.001	− 34.36	1.0	< 0.001	259.77	6.5	< 0.001	− 0.49	0.01	< 0.001	5.98		0.2	< 0.001
Spirometry_base_ (ml)				0.80	0.007	< 0.001				0.77	0.007	< 0.001				0.87		0.008	< 0.001
Spirometry_base_ (ml) * TIC				− 0.06	0.001	< 0.001				− 0.07	0.001	< 0.001				− 0.07		0.002	< 0.001
TWEI	− 21.76	7.3	0.003	− 22.04	5.4	< 0.001	− 28.71	7.6	< 0.001	− 20.53	6.1	< 0.001	0.07	0.1	0.415	− 0.14		0.1	0.044
TWEI * TIC				2.00	1.0	0.052				3.78	1.2	0.002				− 0.03		0.01	0.031
Years of dust exposure (yr)	− 1.31	1.5	0.383	3.12	0.9	< 0.001	− 2.22	1.6	0.155	4.27	1.0	< 0.001	0.02	0.02	0.252	− 0.002		0.01	0.890
Years of dust exposure * TIC				− 1.43	0.1	< 0.001				− 1.79	0.1	< 0.001			− 0.002	0.001		0.044	

*SE*  standard error of mean, *BMI*  body mass index, *TIC*  time in cohort, *FEV1*  forced expiratory volume in 1 s, *FVC*  forced vital capacity, *TWEI*  time-weighted exposure intensity

### Occupational dust exposure and longitudinal data of WBC count and its differential

Current smokers, packyears and years of dust exposure in males (Table 3), and BMI in either sexes (Table 3 and Additional file 1: Table S6) were associated with higher WBC count and all differential cell counts (neutrophils, lymphocytes. and mid-range absolute counts), consistent with the fact that cigarette smoking, higher BMI, and years of dust exposure increased systemic inflammation. However, age and TIC were associated with lower levels of WBC count and most differential counts in either sexes, suggesting aging may reduce general immunity. Interestingly, there was no evidence supporting TWEI associated with increased WBC count and differential cell counts in either sexes. In opposite, TWEI was associated with lower lymphocyte count in males (Table 3) and with lower mid-range absolute count (MID) count in either sexes (Table 3 and Additional file 1: Table S6). Self-reported exposure to toxic gases (e.g., carbon monoxide, nitrogen monoxide and dioxide, and benzene and its derivatives) was not associated with WBC count and its differential (data not shown).

**Table 3:** The association between dust exposure and white blood cell count and its differential in male workers using linear mixed-effects model (n = 6100)

Variable	WBC (10^9^ cells per L)			NEU (10^9^ cells per L)			LYM (10^9^ cells per L)			MID (10^9^ cells per L)		
	β	SE	P	β	SE	P	β	SE	P	β	SE	P
Intercept	7.0064	0.567	< 0.001	4.5810	0.457	< 0.001	2.1658	0.197	< 0.001	0.2124	0.037	< 0.001
Age (year)	− 0.0284	0.005	< 0.001	− 0.0156	0.004	< 0.001	− 0.0126	0.002	< 0.001	− 0.0004	0.000	0.236
Current smoker	0.2943	0.035	< 0.001	0.2174	0.030	< 0.001	0.0862	0.012	< 0.001	0.0137	0.003	< 0.001
Packyears (py)	0.0227	0.002	< 0.001	0.0178	0.002	< 0.001	0.0045	0.001	< 0.001	0.0010	0.0002	< 0.001
BMI (kg/m^2^)	0.0758	0.005	< 0.001	0.0552	0.004	< 0.001	0.0188	0.002	< 0.001	0.0023	0.0003	< 0.001
Height (m)	− 0.0124	0.003	< 0.001	− 0.0103	0.003	< 0.001	− 0.0019	0.001	0.083	− 0.0001	0.0002	0.686
TIC (yr)	− 0.0409	0.003	< 0.001	− 0.0250	0.002	< 0.001	− 0.0099	0.001	< 0.001	− 0.0053	0.0003	< 0.001
TWEI	− 0.0108	0.024	0.653	0.0270	0.019	0.164	− 0.0312	0.008	< 0.001	− 0.0075	0.002	< 0.001
Years of dust exposure (year)	0.0175	0.005	< 0.001	0.0121	0.004	0.003	0.0045	0.002	0.008	0.0008	0.000	0.018

*TWEI*  time-weighted exposure intensity, *BMI*  body mass index, *TIC*  time in cohort, *WBC*  white blood count, *NEU*  neutrophilicgranulocyte, *LYM*  lymphocyte, *MID* mid-range absolute count including monocytes, eosinophils and basophils, *SE*  standard error of mean

### Associations of WBC count and its differential with a spirometric decline

Higher baseline WBC count and differential (neutrophil and lymphocyte) counts were associated with lower longitudinal measurements and a more rapid decline of FEV1 and FVC in male workers only (Table 4). The MID count was associated with a more rapid decline of FEV1 and FVC in male workers only (Table 4). Lymphocyte count was associated with lower longitudinal measurements of FEV1 and FVC but not with a decline in females (Table 4). We further stratified workers into quartiles based on WBC count or differential counts to characterize their dose–response relationship with longitudinal lung function measurements and their declines (Additional file 1: Table S7). Higher WBC count and differential (neutrophil and lymphocyte count) were monotonically associated with lower longitudinal measurements and a more rapid decline of FEV1 and FVC in males only (Additional file 1: Table S7 and Additional file 1: Figure S1). MID was monotonically associated with a more rapid decline of FEV1 and FVC in male workers only (Additional file 1: Table S7 and Figure S1). Lymphocyte count was associated with lower longitudinal measurements of FEV1 and FVC in a dose–response manner in females (Additional file 1: Table S7).

**Table 4:** The sex-specific association between white blood cell count and its differential count (analyzed as continuous variables) at baseline and spirometry using linear mixed-effects model ^a^

Variable	Model ^b^	FEV1 (ml/s)						FVC (ml)						FEV1/FVC (%)					
		Male (n = 5949)			Female (n = 1290)			Male (n = 5949)			Female (n = 1290)			Male (n = 5949)			Female (n = 1290)		
		β	SE	P	β	SE	P	β	SE	P	β	SE	P	β	SE	P	β	SE	P
WBC	1	− 38.09	6.4	< 0.001	− 11.88	10.3	0.249	− 37.29	6.7	< 0.001	− 12.65	11.0	0.249	− 0.14	0.1	0.089	− 0.07	0.2	0.692
WBC * TIC	2	− 4.33	0.9	< 0.001	1.24	1.7	0.455	− 4.62	1.1	< 0.001	1.31	1.9	0.490	− 0.02	0.01	0.128	− 0.002	0.03	0.923
NEU	1	− 33.04	6.1	< 0.001	− 6.62	9.9	0.504	− 31.50	6.4	< 0.001	− 5.89	10.5	0.576	− 0.13	0.1	0.083	− 0.10	0.2	0.546
NEU * TIC	2	− 3.46	0.9	< 0.001	1.23	1.6	0.436	− 3.58	1.0	< 0.001	1.47	1.8	0.417	− 0.02	0.01	0.082	− 0.006	0.03	0.831
LYM	1	− 30.30	6.7	< 0.001	− 17.55	7.9	0.026	− 30.33	7.0	< 0.001	− 17.46	8.4	0.037	− 0.10	0.1	0.248	− 0.08	0.1	0.542
LYM * TIC	2	− 3.96	1.0	< 0.001	0.20	1.3	0.880	− 4.53	1.1	< 0.001	0.06	1.5	0.970	− 0.002	0.01	0.859	− 0.001	0.02	0.954
MID	1	3.80	3.7	0.300	11.63	14.1	0.410	0.88	3.8	0.817	4.61	15.0	0.759	0.06	0.0	0.187	0.21	0.2	0.369
MID * TIC	2	− 1.54	0.5	0.003	− 1.03	2.3	0.647	− 1.18	0.6	0.046	− 1.94	2.6	0.452	− 0.01	0.007	0.063	0.02	0.04	0.576

*WBC*  white blood count, *NEU*  neutrophilicgranulocyte, *LYM*  lymphocyte, *MID*  mid-range absolute count including monocytes, eosinophils and basophils, *SE*  standard error of mean, *TIC*  time in cohort

^a^β and standard error of mean was calculated using the interquartile range of WBC count and its differential at baseline as the unit of change. Inter-quartile ranges in male workers: WBC: 2.0*10^9^/L; NEU: 1.6*10^9^/L; LYM: 0.7*10^9^/L; MID: 0.1*10^9^/L. Inter-quartile ranges in female workers: WBC: 1.8*10^9^/L; NEU: 1.4*10^9^/L; LYM: 0.5*10^9^/L; MID: 0.2*10^9^/L

^b^Model 1 assessed the associations of longitudinal lung function measurements as the outcome with baseline WBC and its differential with adjustment for age, smoking status (male only), packyears (male only), height, BMI, TIC, TWEI, and years of dust exposure. Model 2 assessed the associations of baseline WBC and its differential on lung function decline and used longitudinal lung function measurements as the outcome. In addition to all covariates adjusted in model 1, model 2 also included baseline spirometry and baseline WBC and its differential and interaction terms of TIC with TWEI, baseline spirometry, years of dust exposure, and baseline WBC and its differential. A negative β of the interaction term between TIC and baseline WBC in model 2 indicated that higher baseline WBC was associated with a more rapid decline of lung function

We further tested whether elevation of WBC count and its differential post-baseline, as an indicator for the elevation of systemic inflammation over time was associated with a more rapid decline of lung function. Change in WBC and its differential for each visit relative to baseline level were calculated and were included in the linear mixed-effects models together with its interaction of TIC. Interestingly, the elevation of WBC and its differential (e.g., neutrophil and MID) was associated with a more rapid decline of FEV1 and FVC in either sexes (Table 5). Moreover, the magnitude of association seems to be more robust in female than male workers. Elevation of lymphocyte count was associated with a more rapid FEV1 decline in male workers only, resulted in a more rapid decline of FEV1/FVC ratio.

**Table 5:** The association between the increase of white blood cell count and its differential post-baseline and lung function decline assessed using a linear mixed-effects model

Variable ^a^	FEV1 (ml/s)						FVC (ml)						FEV1/FVC (%)					
	Male (n = 5864)			Female (n = 1260)			Male (n = 5864)			Female (n = 1260)			Male (n = 5864)			Female (n = 1260)		
	β	SE	P	β	SE	P	β	SE	P	β	SE	P	β	SE	P	β	SE	P
Change of WBC *TIC	− 0.90	0.2	< 0.0001	− 1.18	0.3	< 0.0001	− 0.80	0.2	< 0.0001	− 1.34	0.4	< 0.0001	− 0.004	0.002	0.050	0.001	0.005	0.840
Change of NEU * TIC	− 0.71	0.2	< 0.0001	− 0.96	0.3	0.001	− 0.75	0.2	< 0.0001	− 0.99	0.3	0.004	− 0.001	0.002	0.455	− 0.003	0.005	0.611
Change of LYM *TIC	− 0.43	0.2	0.006	− 0.24	0.3	0.448	− 0.05	0.2	0.790	− 0.49	0.4	0.177	− 0.009	0.002	< 0.0001	0.006	0.005	0.269
Change of MID * TIC	− 1.03	0.1	< 0.0001	− 1.37	0.3	< 0.0001	− 1.14	0.1	< 0.0001	− 1.62	0.3	< 0.0001	− 0.0003	0.002	0.834	0.002	0.004	0.658

*WBC*  white blood count, *NEU*  neutrophilicgranulocyte, *LYM*  lymphocyte, *MID*  mid-range absolute count including monocytes, eosinophils and basophils, *SE*  standard error of mean, *TIC*  time in cohort

^a^Delta change was calculated as values of WBC and its differential for each non-baseline visit minus baseline value for each individual and was included in the model for assessing its association with lung function decline. The model used longitudinal lung function measurements as the outcome and included age, smoking status (male only), packyears (male only), height, BMI, TIC, TWEI, years of dust exposure, baseline spirometry, baseline WBC and its differential, delta change of WBC and its differential, and interaction terms of TIC with TWEI, baseline spirometry, years of dust exposure, baseline WBC and its differential, and delta change of WBC and its differential. A negative β of the interaction term between TIC and delta change of WBC indicated that an increase in WBC post-baseline was associated with a more rapid decline of lung function. β and standard error of the mean was calculated using the interquartile range as the unit of change. Inter-quartile ranges for delta changes were calculated based on the distribution of annual changes of WBC (i.e., last WBC – baseline WBC / TIC) and its differential in cohort members with positive delta change values (n = 2211, 2360, 2235, and 1355 for WBC, NEU, LYM, and MID, respectively). WBC: 0.187*10^9^/L; NEU: 0.150*10^9^/L; LYM: 0.060*10^9^/L; MID: 0.014*10^9^/L. The inter-quartile range values were not sex-specific because we want to quantitatively compare the magnitude of associations between sexes

### Sensitivity analyses

We are concerned that the associations between occupational dust exposure or WBC count and differential and lung function decline could be potentially confounded by cigarette smoking. None of these three-way interaction terms on current smoker × TWEI × TIC or current smoker × WBC × TIC were statistically significant (data not shown). We also repeated the main analyses in subjects (n = 6735) with two or more visits, findings similar to that seen in the entire 7513 workers were identified. In addition, we calculated the time-weighted exposure intensity (TWEI) based on self-reported dust exposure history up to every medical surveillance and observed the same trend for the associations between dust exposure, WBCs and spirometric decline. (data not shown).

## Discussion

In this longitudinal cohort of 7513 workers, occupational exposure to inhalable particles containing metal elements prior to baseline evaluation was inversely associated with longitudinal measurements of FEV1 and FVC over a follow-up period of 10 years. We observed that cohort members with high levels of baseline WBC counts and differential had lower longitudinal measurements and a more rapid decline of FEV1 and FVC in male workers in a dose-dependent manner. Moreover, the elevation of WBC counts and its differential count post-baseline as an indicator of increasing systemic inflammation over time was associated with a more rapid decline of FEV1 and FVC in either sexes. Finally, sex disparity of the associations between dust exposure, WBC, and lung function was identified with stronger adverse effects of dust exposure or change of WBC seen in females as well as stronger effects of baseline WBC seen in males. The mechanisms underlying the sex disparity may be attributed to differences in dust exposure level [24], inherited differences in lung structure [25], or differences in sex hormones regulating lung homeostasis upon the challenge of dust exposure.

Occupational exposure to inhalable dust in the iron and steel industry has been shown to compromise lung function and increase the risk of chronic airflow obstruction in exposed workers [1, 2, 9, 10]. In this study, the decline rates of FEV1 and FVC are much faster than those observed in current and former cigarette smokers from a NM, USA-based Lovelace Smokers cohort with a median packyears of 36.0 and relatively healthy lung function, but comparable to smokers from Pittsburgh Lung Screening Study cohort with a median packyears of 59 and much-compromised lung function [26] and from Lung Health Study with all participants having mild to moderate airway obstruction [27], supporting the adverse effects of occupational dust exposure on the age-related decline of spirometry. A case–control study from New York City firefighters has shown that inhalable dust could remain in the lungs and be pro-inflammatory for up to 10 months after cessation of fire smoke exposure [28, 29]. However, we did not see an accelerated FEV1 and FVC decline associated with dust exposure prior to baseline. This finding was not altered even when dust exposure calculated up to each physical examination date was included in the model. Such patterns of results were supported by a most recent study in which prior airborne occupational exposures (e.g., biological dust, mineral dust, gases/fumes, insecticides, herbicides, fungicides, aromatic, chlorinated, other solvents, and metals) were associated with lower lung function measurements but not with annual decline using the Lifelines Cohort Study [30]. This could be explained by that most workers in our study have been working at a job title with occupational exposures for more than a decade prior to the baseline visit. Thus, workers may already develop resistance or saturation, a state of indifference or non-reactivity towards a substance that would normally be expected to excite a more exaggerated health effect [30]. A second reasonable explanation is that workers who are sensitive to dust-induced health effects could have switched their job with lower dust exposure or the pre-employment selection criteria could select subjects who are less likely to be affected by occupational dust exposure on lung function decline. Indeed, we did find that workers at occupational positions with higher dust exposure tend to be taller than workers from other positions and height is associated with better lung function and greater resilience for age-related lung function decline.

Higher WBC and its differential at baseline were associated with lower longitudinal measurements of lung function and a higher decline rate of FEV1 and FVC among the male workers. Baseline WBC counts implied the overall levels of inflammation and the post-baseline WBC counts change reflected the increase of system inflammation over time. Besides, this association is independent of occupational dust exposure. Furthermore, although on average WBC and its differential declined over time, the elevation of WBC and its differential post-baseline was associated with a more rapid decline of FEV1 and FVC in either sexes as well. These findings strongly support our hypothesis that elevated systemic inflammatory markers predict a more rapid decline of lung function in workers [11, 31, 32]. However, the temporal relationship between baseline WBC and its differential and subsequent lung function change and the compelling dose–response relationship does not directly support a causal relationship between systemic inflammation and lung function impairment. Instead, it may reflect a latent condition that workers with higher systemic inflammation are more vulnerable to dust exposure-induced health effects. The lack of a positive correlation between TWEI and WBC and its differential also suggested this latent inflammatory condition is more reflective of individual predisposition rather than an acquired trait due to chronic iron and steel dust exposure. Predisposition is an increased likelihood of developing a particular disease based on a person's genetic makeup rather than acquired character. The magnitude of associations of lung function or its decline with WBC and its differential is very comparable and this does not support a more dominant effect of certain leucocytes. Thus, all these suggest systemic inflammation as a holistic readout of individual predisposition that well predicts the severity of pulmonary toxicity caused by dust exposure containing metals in workers [8, 11].

We also found that elevation of post-baseline lymphocyte count was associated with a more rapid decline of FEV1/FVC ratio in male workers only. The ratio of FEV1 to FVC measures the amount of air a person can forcefully exhale in one second relative to the total amount of air individuality can exhale, which is the established index for diagnosing airway obstruction [33]. This ratio is decreased in obstructive lung disorders and normal in restrictive lung disorders. Research on COPD patients and healthy participants found higher levels of lymphocytes in COPD patients than the control group [34]. Chronic obstructive pulmonary disease is associated with inflammation of airway epithelium, including an increase in the number of intraepithelial T cells. Increased apoptosis of these T cells may result in unbalanced cellular homeostasis, defective clearance of apoptotic material by monocytes/macrophages, secondary necrosis, and prolongation of the inflammatory response. The increased T-cell death may be associated with the upregulation of apoptotic pathways, TGF-beta, TNF-alpha, and Fas in the peripheral blood [35].

This present study benefits greatly from a large sample size, high-quality data for longitudinal measurements of lung spirometry and WBC, and a long follow-up period that together contributes to sufficient statistical power and a more precise assessment of the magnitude of associations. However, this study does have limitations. Due to the lack of reliable occupational monitoring data for all years except 2006 and 2007, for many job posts, and the low exposure category, the job-exposure matrix could not be established for studied subjects. Second, we do not have the resources to assess what has happened in the first ten years of employment of the enrolled workers. A cohort with careful and repeated assessment of occupational exposure would help to disentangle the relationship between dust exposure, systemic inflammation, and the initial decline of lung function.

## Conclusion

In conclusion, we have shown that previous occupational exposure to inhalable dust-containing metals resulted in a reduction in FEV1 and FVC. Higher WBC and its differential at baseline or elevation post-baseline were associated with a more rapid decline of FEV1 and FVC. Future studies should focus on a more quantitative assessment of occupational exposure and its constituents, a cohort of new employees to detect early changes of health effects after exposure initiation, and novel local and systemic inflammation biomarkers [36].

## Supplementary Information

**Additional file 1: Table S1.** Inhalable dust concentration (mg/m^3^) for different exposure category. **Table S2.** The association betwwen dust exposure and spirometry in female workers using linear mixeds-effects model (n = 1295). **Table S3.** The univariate analyses assessing the associations between time-weighted exposure intensity and characteristic variables as the outcomes. **Table S4.** The influence of inclusion of height and TIC interaction on the estimates and P values for TWEI and TIC interaction in male workers using linear mixed-effects model (n = 6100). **Table S5.** The influence of inclusion of height and TIC interaction on the estimates and P values for TWEI and TIC interaction in female workers using linear mixed-effects model (n = 1295). **Table S6.** The association between dust exposure and white blood cell count and its differential in female workers using linear mixed-effects model (n = 1295). **Table S7.** The association between categorized white blood cell count and its differential at baseline on longitudinal spirometry using linear mixed-effects model to assess the dose–response relationship. **Figure S1.** Dose–response relationship between WBC count and its differential and FEV1 decline in workers by sex. Baseline WBC count and its differential were converted into categorical variables based on sex-specific 25th percentile, median, and 75th percentile (see Table S7 for range limits). Linear mixed-effects model assessed the of quartile group status and FEV1 decline with Q1 as the reference. Q1, Q2, Q3, and Q4 group status was coded as numerical 0, 1, 2, and 3 in the trend test. The detailed modeling strategy was introduced in the footnote of Table S7. A significant dose–response relationship was identified for WBC, NEU, LYM, and MID in male workers only. A more rapid FEV1 decline was identified in male workers with higher levels of WBC or its differential. Only three groups for MID in males were available due to the discrete distribution of the MID data. A statistically significant test results (*) means P < 0.05 compared to the Q1 group.

## Data Availability

The datasets used and/or analyzed during the current study are available from the corresponding author on reasonable request. The datasets generated and/or analyzed during the current study are not publicly available but are available from the corresponding author on reasonable request.

## Abbreviations

FEV1: Forced expiratory volume in 1 s
FVC: Forced vital capacity
COPD: Chronic obstructive pulmonary disease
WBC: White blood cell
NEU: Neutrophilic granulocyte
LYM: Lymphocyte
MID: Mid-range absolute count
BMI: Body mass index
TWEI: Time-weighted exposure intensity
ATS: American Thoracic Society
ERS: European Respiratory Society standards
TIC: Time in cohort
TWA: Time-weighted average
SE: Standard error of mean
M: Mean
SD: Standard deviation
Q: Quartile
