# Supplementary material for: Blood leukocyte count as a systemic inflammatory biomarker associated with a more rapid spirometric decline in a large cohort of iron and steel industry workers
Source: Respir Res. 2021 Sep 26;22:254. doi: 10.1186/s12931-021-01849-y (PMC8467242; doi:10.1186/s12931-021-01849-y)

**Additional methods, tables, and figure**

**Blood leukocyte count as a systemic inflammatory biomarker associated with a more rapid decline of spirometry in a large cohort of iron and steel industry workers**

Nan Kong^1^*, Guoshun Chen^2^*, Haitao Wang^1^, Jianyu Li^1^, Shuzhen Yin^2^, Xue Cao^1^, Tao Wang^1^, Xin Li^1^, Yanan Li^1^, Huanling Zhang^2^, Shanfa Yu^3^, Jinglong Tang^1^, Akshay Sood^4^, Yuxin Zheng^1#^, Shuguang Leng^4,5,1#^

**Method**

**Study subjects**

This longitudinal study was conducted on 7,575 workers who were required to participate in the Worker Health Surveillance program at Wugang Institute for Occupational Health between 2008 and 2017. These workers worked in workshops with known occupational hazards including dust containing metal elements, noise, hazardous gases such as carbon monoxide and nitrogen monoxide and dioxide, heat, visual stress, and power frequency electric field at Wuyang Iron and Steel Company Limited, Hangang Group in Henan, China (plus code: 7FGW+WX). This company has been using electric arc furnaces steelmaking technique to manufacture steel from scrap or direct reduced iron melted by electric arcs and mainly has steel making, continuous casting, rolling, and oxygen-making plants. According to the Reports of Occupational Hazard Control Assessment conducted by Henan Institute of Occupational Medicine in 2006 and 2007, inhalable dust and noise are the two occupational hazards that have samples exceeding the national standards (the permissible concentration time-weighted average of 8 mg/m3 for inhalable dust and 85 dB(A) for noise in China). The start year of this longitudinal analysis was determined by the availability of complete archived medical data. This cohort was dynamic with workers entering and leaving the cohort at different times. In general, individual participants in this program received medical assessment every other year. Medical surveillance workout was recommended by the China Ministry of Health. Working history, occupational exposure history, medical history and symptoms, general physical exams, complete blood count (CBC), comprehensive metabolic panel, urine routine, chest X-ray, electrocardiogram, audiogram, and spirometry were collected. The de-identified data were obtained from Wugang Institute for Occupational Health. The Research Ethics Committee of the Qingdao University School of Medicine approved the study protocol. The Committee determined that consenting was not necessary.

Table S1. Inhalable dust concentration (mg/m^3^) for different exposure category ^a^

| Year | Exposure category | No. posts assessed | M ± SD | Mdn | Min | Max |
| --- | --- | --- | --- | --- | --- | --- |
| 2006 | Low | Not sampled |  |  |  |  |
|  | Medium | 21 | 1.13 ± 0.65 | 0.91 | 0.21 | 2.3 |
|  | High | 14 | 8.27 ± 4.82 | 7.45 | 2.5 | 15.6 |
| 2007 | Low | Not sampled |  |  |  |  |
|  | Medium | 16 | 2.01 ± 1.51 | 1.45 | 0.36 | 4.7 |
|  | High | 16 | 12.70 ± 7.13 | 10.35 | 5.1 | 28.3 |

Deﬁnition of abbreviations: M = mean; SD = standard deviation; Mdn = median; Min = minimum; Max = maximum

^a^ There were 329 repeated job titles of the 79 workshops and one type of work may distribute in different workshops. The workshop is a relatively enclosed environment, there was little difference between different types of work in exposure levels in the same workshop. Based on the percentage of subjects reporting dust exposure, consultation with the occupational hygienists, historical personal sampling data, and epidemiological consideration of sample size within each exposure category, seventy-nine workshops were classified into low (n=43), medium (n=20), and high (n=16) exposure categories to maximize the statistical power of the study. Inhalable dust concentration was measured in 10 workshops in 2006 according to GB5748-85 and 8 workshops in 2007 according to GBZ2-2002. Eight-hour time-weighted average airborne concertation of dust at breathing zone was assessed using a personal air sampler. Six out of 35 air samples from 2006 and 12 out of 32 air samples from 2007 exceeded the permissible concentration time-weighted average of 8 mg/m^3^. Moreover, ninety percent of dust mass was non-Silicon dioxide (potentially as metal dust). Size distribution analysis found over 80% particles had sizes less than 5 μm.

Table S2. The association betwwen dust exposure and spirometry in female workers using linear mixeds-effects model (n=1295)

| Variable | | FEV1 (ml/s) | | | | | |  | FVC (ml) | | | | | |  | FEV1/FVC (%) | | | | | |
| --- | --- | --- | --- | --- | --- | --- | --- | --- | --- | --- | --- | --- | --- | --- | --- | --- | --- | --- | --- | --- | --- |
|  |  | Model 1 | | | Model 2 | | |  | Model 1 | | | Model 2 | | |  | Model 1 | | | Model 2 | | |
|  |  | β | SE | P | β | SE | P |  | β | SE | P | β | SE | P |  | β | SE | P | β | SE | P |
| Intercept | | -707.1 | 266.7 | 0.008 | -1217 | 158.5 | <.001 |  | -1228 | 283 | <.001 | -1619 | 177.9 | <.001 |  | 102.09 | 4.3 | <.001 | 17.96 | 3.2 | <.001 |
| Age (yr) | | -19.55 | 2.9 | <.001 | -4.92 | 1.8 | 0.005 |  | -18.15 | 3.1 | <.001 | -3.89 | 2.0 | 0.048 |  | -0.11 | 0.1 | 0.024 | -0.07 | 0.03 | 0.017 |
| Current smoker | | NC | NC | NC | NC | NC | NC |  | NC | NC | NC | NC | NC | NC |  | NC | NC | NC | NC | NC | NC |
| Packyears (py) | | NC | NC | NC | NC | NC | NC |  | NC | NC | NC | NC | NC | NC |  | NC | NC | NC | NC | NC | NC |
| BMI (kg/m^2^) | | -4.52 | 2.6 | 0.084 | 1.49 | 1.6 | 0.335 |  | -2.85 | 2.8 | 0.305 | 0.09 | 1.7 | 0.959 |  | -0.06 | 0.04 | 0.133 | 0.05 | 0.03 | 0.088 |
| Height (cm) | | 26.82 | 1.5 | <.001 | 11.91 | 1.0 | <.001 |  | 31.26 | 1.6 | <.001 | 15.21 | 1.1 | <.001 |  | -0.04 | 0.03 | 0.151 | -0.03 | 0.02 | 0.098 |
| TIC (yr) | | -39.06 | 1.4 | <.001 | 173.69 | 9.5 | <.001 |  | -26.66 | 1.6 | <.001 | 212.4 | 10.9 | <.001 |  | -0.5 | 0.02 | <.001 | 6.73 | 0.3 | <.001 |
| Spirometry_base_ (ml) | |  |  |  | 0.79 | 0.01 | <.001 |  |  |  |  | 0.77 | 0.02 | <.001 |  |  |  |  | 0.86 | 0.02 | <.001 |
| Spirometry_base_ (ml) * TIC | |  |  |  | -0.07 | 0.003 | <.001 |  |  |  |  | -0.07 | 0.003 | <.001 |  |  |  |  | -0.08 | 0.004 | <.001 |
| TWEI |  | -41.96 | 10.8 | <.001 | -12.79 | 8.2 | 0.117 |  | -36.67 | 11.5 | 0.001 | -10.75 | 9.2 | 0.245 |  | -0.31 | 0.2 | 0.079 | -0.14 | 0.1 | 0.318 |
| TWEI * TIC | |  |  |  | 3.57 | 1.7 | 0.033 |  |  |  |  | 4.18 | 1.9 | 0.03 |  |  |  |  | -0.03 | 0.03 | 0.328 |
| Years of dust exposure (yr) | | 2.57 | 2.6 | 0.326 | 1.5 | 1.6 | 0.357 |  | 2.11 | 2.8 | 0.499 | 0.46 | 1.8 | 0.801 |  | 0.02 | 0.04 | 0.667 | 0.06 | 0.03 | 0.04 |
| Years of dust exposure * TIC | |  |  |  | -1.39 | 0.2 | <.001 |  |  |  |  | -1.48 | 0.2 | <.001 |  |  |  |  | -0.006 | 0.003 | 0.046 |

Definition of abbreviations: SE = standard error of mean; BMI = body mass index; TIC = time in cohort; FEV1 = forced expiratory volume in one second; FVC = forced vital capacity; TWEI = time-weighted exposure intensity

Table S3. The univariate analyses assessing the associations between time-weighted exposure intensity and characteristic variables as the outcomes

| Variable ^a^ | TWEI | | | | | | |
| --- | --- | --- | --- | --- | --- | --- | --- |
|  | Male | | |  | Female | | |
|  | β | 95%CI | P |  | β | 95%CI | P |
| Age (yr) | -3.861 | (-4.154, -3.569) | <.001 |  | -2.007 | (-2.547, -1.466) | <.001 |
| BMI (kg/m^2^) | -0.605 | (-0.727, -0.483) | <.001 |  | -0.184 | (-0.412, 0.043) | 0.113 |
| Years of dust exposure (yr) | -3.834 | (-4.146, -3.523) | <.001 |  | -1.929 | (-2.520, -1.339) | <.001 |
| Height (cm) | 0.631 | (0.444, 0.817) | <.001 |  | 0.985 | (0.605, 1.365) | <.001 |
| Current smoker | 0.966 | (0.903, 1.033) | 0.312 ^b^ |  | NC | NC | NC |
| Packyears (py) | -1.548 | (-1.853, -1.243) | <.001 |  | NC | NC | NC |
| HGB (g/L) | 0.683 | (0.2554, 1.140) | 0.003 |  | 0.845 | (-0.170, 1.859) | 0.103 |

Definition of abbreviations: BMI = body mass index; HGB = hemoglobin; TWEI = time-weighted exposure intensity

^a^ Sample size: 6188 males and 1325 females for age, years of dust exposure, current smoker (male only), and packyears (male only). 6110 males and 1295 females for BMI and height. 5035 males and 1174 females for HGB.

^b^ Logistic regression, e^β^, 95%CI, and p values were presented.

Table S4. The influence of inclusion of height and TIC interaction on the estimates and P values for TWEI and TIC interaction in male workers using linear mixed-effects model (n=6100)

| Variable | FEV1 (ml/s) | | |  | FVC (ml) | | |  | FEV1/FVC (%) | | |
| --- | --- | --- | --- | --- | --- | --- | --- | --- | --- | --- | --- |
|  | β | SE | P |  | β | SE | P |  | β | SE | P |
| Intercept | -219.54 | 124.1 | 0.077 |  | -356.40 | 140.4 | 0.011 |  | 12.75 | 1.8 | <.0001 |
| Age (yr) | -7.82 | 1.0 | <.0001 |  | -9.32 | 1.1 | <.0001 |  | -0.01 | 0.01 | 0.426 |
| Current smoker | -1.87 | 7.3 | 0.797 |  | 3.59 | 8.2 | 0.661 |  | -0.11 | 0.1 | 0.239 |
| Packyears (py) | -0.75 | 0.5 | 0.107 |  | -0.87 | 0.5 | 0.094 |  | 0.005 | 0.006 | 0.430 |
| BMI (kg/m^2^) | -0.51 | 0.9 | 0.555 |  | -2.72 | 1.0 | 0.005 |  | 0.03 | 0.01 | 0.003 |
| TIC (yr) | -185.41 | 23.3 | <.0001 |  | -303.13 | 27.1 | <.0001 |  | 7.47 | 0.3 | <.0001 |
| Height (cm) | 6.51 | 0.7 | <.0001 |  | 8.67 | 0.8 | <.0001 |  | -0.01 | 0.009 | 0.274 |
| Height (cm) * TIC | 2.41 | 0.1 | <.0001 |  | 3.55 | 0.2 | <.0001 |  | -0.008 | 0.002 | <.0001 |
| Spirometry_base_ (ml) | 0.83 | 0.007 | <.0001 |  | 0.81 | 0.007 | <.0001 |  | 0.87 | 0.008 | <.0001 |
| Spirometry_base_ (ml) * TIC | -0.07 | 0.001 | <.0001 |  | -0.08 | 0.001 | <.0001 |  | -0.07 | 0.002 | <.0001 |
| TWEI | -19.13 | 5.3 | 0.0003 |  | -15.36 | 6.0 | 0.011 |  | -0.15 | 0.1 | 0.029 |
| TWEI * TIC | 0.48 | 1.0 | 0.641 |  | 1.33 | 1.2 | 0.265 |  | -0.02 | 0.01 | 0.083 |
| Years of dust exposure (yr) | 3.18 | 0.9 | 0.001 |  | 4.34 | 1.0 | <.0001 |  | 8.9E-6 | 0.01 | 0.999 |
| Years of dust exposure (yr) * TIC | -1.44 | 0.1 | <.0001 |  | -1.78 | 0.1 | <.0001 |  | -0.003 | 0.001 | 0.010 |

Definition of abbreviations: SE = standard error of mean; BMI = body mass index; TIC = time in cohort; FEV1 = forced expiratory volume in one second; FVC = forced vital capacity; TWEI = time-weighted exposure intensity

Table S5. The influence of inclusion of height and TIC interaction on the estimates and P values for TWEI and TIC interaction in female workers using linear mixed-effects model (n=1295)

| Variable | FEV1 (ml/s) | | |  | FVC (ml) | | |  | FEV1/FVC (%) | | |
| --- | --- | --- | --- | --- | --- | --- | --- | --- | --- | --- | --- |
|  | β | SE | P |  | β | SE | P |  | β | SE | P |
| Intercept | -344.21 | 194.8 | 0.078 |  | -369.32 | 220.1 | 0.094 |  | 13.24 | 3.7 | 0.0004 |
| Age (yr) | -4.90 | 1.7 | 0.005 |  | -3.85 | 1.9 | 0.048 |  | -0.07 | 0.03 | 0.018 |
| Current smoker | NC | NC | NC |  | NC | NC | NC |  | NC | NC | NC |
| Packyears (py) | NC | NC | NC |  | NC | NC | NC |  | NC | NC | NC |
| BMI (kg/m^2^) | 1.45 | 1.5 | 0.346 |  | 0.004 | 1.7 | 0.998 |  | 0.05 | 0.03 | 0.084 |
| TIC (yr) | -120.03 | 39.6 | 0.003 |  | -205.34 | 45.2 | <.0001 |  | 8.30 | 0.7 | <.0001 |
| Height (cm) | 6.05 | 1.2 | <.0001 |  | 6.78 | 1.4 | <.0001 |  | 0.004 | 0.02 | 0.860 |
| Height (cm) * TIC | 1.97 | 0.3 | <.0001 |  | 2.80 | 0.3 | <.0001 |  | -0.01 | 0.004 | 0.017 |
| Spirometry_base_ (ml) | 0.81 | 0.02 | <.0001 |  | 0.80 | 0.02 | <.0001 |  | 0.86 | 0.02 | <.0001 |
| Spirometry_base_ (ml) * TIC | -0.08 | 0.003 | <.0001 |  | -0.08 | 0.003 | <.0001 |  | -0.08 | 0.004 | <.0001 |
| TWEI | -6.05 | 8.2 | 0.459 |  | -1.12 | 9.2 | 0.903 |  | -0.17 | 0.1 | 0.232 |
| TWEI * TIC | 0.98 | 1.7 | 0.565 |  | 0.53 | 1.9 | 0.785 |  | -0.02 | 0.03 | 0.557 |
| Years of dust exposure (yr) | 1.38 | 1.6 | 0.393 |  | 0.22 | 1.8 | 0.904 |  | 0.06 | 0.03 | 0.034 |
| Years of dust exposure (yr) * TIC | -1.35 | 0.2 | <.0001 |  | -1.40 | 0.2 | <.0001 |  | -0.01 | 0.003 | 0.027 |

Definition of abbreviations: SE = standard error of mean; Q = quartile; BMI = body mass index; TIC = time in cohort; FEV1 = forced expiratory volume in one second; FVC = forced vital capacity

Table S6. The association between dust exposure and white blood cell count and its differential in female workers using linear mixed-effects model (n=1295)

| Variable | WBC (10^9^ cells per L) | | |  | NEU (10^9^ cells per L) | | |  | LYM (10^9^ cells per L) | | |  | MID (10^9^ cells per L) | | |  |
| --- | --- | --- | --- | --- | --- | --- | --- | --- | --- | --- | --- | --- | --- | --- | --- | --- |
|  | β | SE | P |  | β | SE | P |  | β | SE | P |  | β | SE | P |  |
| Intercept | 7.0135 | 1.059 | <.001 |  | 4.2840 | 0.834 | <.001 |  | 2.4157 | 0.378 | <.001 |  | 0.2983 | 0.066 | <.001 |  |
| Age (yr) | -0.0420 | 0.012 | <.001 |  | -0.0271 | 0.009 | 0.003 |  | -0.0141 | 0.004 | <.001 |  | -0.0001 | 0.001 | 0.911 |  |
| Current smoker | NC | NC | NC |  | NC | NC | NC |  | NC | NC | NC |  | NC | NC | NC |  |
| Packyears (py) | NC | NC | NC |  | NC | NC | NC |  | NC | NC | NC |  | NC | NC | NC |  |
| BMI (kg/m2) | 0.0967 | 0.010 | <.001 |  | 0.0715 | 0.008 | <.001 |  | 0.0220 | 0.004 | <.001 |  | 0.0033 | 0.001 | <.001 |  |
| Height (m) | -0.0165 | 0.006 | 0.007 |  | -0.0107 | 0.005 | 0.027 |  | -0.0048 | 0.002 | 0.028 |  | -0.0010 | 0.0004 | 0.007 |  |
| TIC (yr) | -0.0266 | 0.006 | <.001 |  | -0.0184 | 0.005 | <.001 |  | -0.0026 | 0.002 | 0.187 |  | -0.0062 | 0.001 | <.001 |  |
| TWEI | -0.0367 | 0.043 | 0.394 |  | -0.0046 | 0.034 | 0.892 |  | -0.0221 | 0.015 | 0.150 |  | -0.0078 | 0.003 | 0.003 |  |
| Years of dust exposure (yr) | 0.0187 | 0.010 | 0.071 |  | 0.0149 | 0.008 | 0.069 |  | 0.0023 | 0.004 | 0.529 |  | 0.0003 | 0.001 | 0.658 |  |

Definition of abbreviations: TWEI = time-weighted exposure intensity; BMI = body mass index; TIC = time in cohort; WBC = white blood count; NEU = neutrophilicgranulocyte; LYM = lymphocyte; MID = mid-range absolute count; SE = standard error of mean

Table S7. The association between categorized white blood cell count and its differential at baseline on longitudinal spirometry using linear mixed-effects model to assess the dose-response relationship

| Variable ^a^ | | | FEV1 (ml/s) | | | | | |  | FVC (ml) | | | | | |  | FEV1/FVC (%) | | | | | |
| --- | --- | --- | --- | --- | --- | --- | --- | --- | --- | --- | --- | --- | --- | --- | --- | --- | --- | --- | --- | --- | --- | --- |
|  |  |  | Male (n=5949) | | | Female (n=1290) | | |  | Male (n=5949) | | | Female (n=1290) | | |  | Male (n=5949) | | | Female (n=1290) | | |
|  |  |  | β | SE | P | β | SE | P |  | β | SE | P | β | SE | P |  | β | SE | P | β | SE | P |
| WBC | Model 1 | Q1 | REF |  |  | REF |  |  |  | REF |  |  | REF |  |  |  | REF |  |  | REF |  |  |
|  |  | Q2 | 1.64 | 13.8 | 0.905 | 12.42 | 21.8 | 0.569 |  | -2.90 | 14.5 | 0.841 | 27.96 | 23.2 | 0.228 |  | 0.13 | 0.2 | 0.437 | -0.44 | 0.4 | 0.210 |
|  |  | Q3 | -26.41 | 14.2 | 0.062 | -26.59 | 21.5 | 0.217 |  | -15.89 | 14.9 | 0.286 | -41.43 | 22.9 | 0.070 |  | -0.29 | 0.2 | 0.102 | 0.30 | 0.3 | 0.388 |
|  |  | Q4 | -74.55 | 14.6 | <.0001 | -30.36 | 22.9 | 0.184 |  | -71.60 | 15.3 | <.0001 | -25.58 | 24.3 | 0.292 |  | -0.30 | 0.2 | 0.099 | -0.31 | 0.4 | 0.406 |
|  |  | P trend | -24.87 | 4.7 | <.0001 | -12.91 | 7.2 | 0.074 |  | -22.48 | 4.9 | <.0001 | -14.73 | 7.7 | 0.055 |  | -0.13 | 0.1 | 0.023 | -0.01 | 0.1 | 0.929 |
|  | Model 2 | Q1 * TIC | REF |  |  | REF |  |  |  | REF |  |  | REF |  |  |  | REF |  |  | REF |  |  |
|  |  | Q2 * TIC | -1.38 | 2.1 | 0.510 | 8.84 | 3.5 | 0.013 |  | -4.26 | 2.4 | 0.082 | 7.74 | 4.1 | 0.057 |  | 0.06 | 0.03 | 0.026 | 0.08 | 0.1 | 0.200 |
|  |  | Q3 * TIC | -3.99 | 2.1 | 0.058 | 1.52 | 3.5 | 0.666 |  | -4.76 | 2.4 | 0.051 | 0.15 | 4.0 | 0.971 |  | -0.01 | 0.03 | 0.848 | 0.06 | 0.1 | 0.340 |
|  |  | Q4 * TIC | -9.66 | 2.1 | <.0001 | 1.95 | 3.7 | 0.594 |  | -10.60 | 2.5 | <.0001 | 1.41 | 4.2 | 0.736 |  | -0.03 | 0.03 | 0.297 | 0.03 | 0.1 | 0.584 |
|  |  | P trend | -3.15 | 0.7 | <.0001 | -0.005 | 1.2 | 0.997 |  | -3.23 | 0.8 | <.0001 | -0.24 | 1.3 | 0.854 |  | -0.02 | 0.01 | 0.085 | 0.01 | 0.01 | 0.602 |
| NEU | Model 1 | Q1 | REF |  |  | REF |  |  |  | REF |  |  | REF |  |  |  | REF |  |  | REF |  |  |
|  |  | Q2 | -8.77 | 13.7 | 0.522 | -17.09 | 21.1 | 0.418 |  | -9.18 | 14.4 | 0.525 | -2.86 | 22.5 | 0.899 |  | 0.005 | 0.2 | 0.978 | -0.52 | 0.3 | 0.130 |
|  |  | Q3 | -24.88 | 13.9 | 0.074 | -32.47 | 22.3 | 0.146 |  | -26.41 | 14.6 | 0.071 | -23.24 | 23.7 | 0.328 |  | -0.01 | 0.2 | 0.959 | -0.48 | 0.4 | 0.189 |
|  |  | Q4 | -83.14 | 14.5 | <.0001 | -30.91 | 22.6 | 0.172 |  | -73.63 | 15.2 | <.0001 | -31.30 | 24.1 | 0.194 |  | -0.46 | 0.2 | 0.011 | -0.16 | 0.4 | 0.668 |
|  |  | P trend | -16.79 | 4.6 | 0.0003 | -10.92 | 7.2 | 0.127 |  | -23.31 | 4.8 | <.0001 | -11.46 | 7.6 | 0.132 |  | -0.13 | 0.1 | 0.020 | -0.05 | 0.1 | 0.693 |
|  | Model 2 | Q1 * TIC | REF |  |  | REF |  |  |  | REF |  |  | REF |  |  |  | REF |  |  | REF |  |  |
|  |  | Q2 * TIC | -1.33 | 2.1 | 0.523 | -0.13 | 3.5 | 0.970 |  | -3.21 | 2.4 | 0.186 | -0.72 | 4.0 | 0.857 |  | 0.02 | 0.03 | 0.445 | -0.01 | 0.1 | 0.920 |
|  |  | Q3 * TIC | -3.55 | 2.1 | 0.086 | -2.74 | 3.6 | 0.448 |  | -4.42 | 2.4 | 0.065 | -3.75 | 4.1 | 0.364 |  | -0.003 | 0.03 | 0.900 | 0.01 | 0.1 | 0.889 |
|  |  | Q4 * TIC | -8.29 | 2.1 | <.0001 | 0.35 | 3.6 | 0.923 |  | -9.15 | 2.5 | 0.0002 | 0.53 | 4.2 | 0.898 |  | -0.04 | 0.03 | 0.159 | -0.01 | 0.1 | 0.862 |
|  |  | P trend | -2.67 | 0.7 | <.0001 | -0.23 | 1.1 | 0.840 |  | -2.85 | 0.8 | 0.0002 | -0.24 | 1.3 | 0.855 |  | -0.01 | 0.01 | 0.125 | -0.002 | 0.02 | 0.931 |
| LYM | Model 1 | Q1 | REF |  |  | REF |  |  |  | REF |  |  | REF |  |  |  | REF |  |  | REF |  |  |
|  |  | Q2 | -34.72 | 13.3 | 0.009 | -14.58 | 21.9 | 0.505 |  | -31.18 | 14.1 | 0.027 | -9.84 | 23.3 | 0.673 |  | -0.12 | 0.2 | 0.468 | -0.14 | 0.4 | 0.703 |
|  |  | Q3 | -40.52 | 13.6 | 0.003 | -30.43 | 21.5 | 0.158 |  | -36.71 | 14.3 | 0.010 | -22.92 | 22.9 | 0.317 |  | -0.18 | 0.2 | 0.287 | -0.34 | 0.3 | 0.337 |
|  |  | Q4 | -51.96 | 14.6 | 0.0004 | -53.12 | 21.5 | 0.014 |  | -52.50 | 15.2 | 0.001 | -44.88 | 22.9 | 0.050 |  | -0.16 | 0.2 | 0.385 | -0.42 | 0.3 | 0.229 |
|  |  | P trend | -25.88 | 4.6 | <.0001 | -17.33 | 6.8 | 0.011 |  | -16.71 | 4.8 | 0.001 | -14.47 | 7.3 | 0.047 |  | -0.06 | 0.1 | 0.320 | -0.15 | 0.1 | 0.189 |
|  | Model 2 | Q1 * TIC | REF |  |  | REF |  |  |  | REF |  |  | REF |  |  |  | REF |  |  | REF |  |  |
|  |  | Q2 * TIC | -4.30 | 2.1 | 0.036 | 0.24 | 3.6 | 0.946 |  | -4.88 | 2.4 | 0.041 | -4.20 | 4.1 | 0.308 |  | -0.01 | 0.03 | 0.79 | 0.13 | 0.06 | 0.03 |
|  |  | Q3 * TIC | -6.14 | 2.0 | 0.002 | -0.07 | 3.6 | 0.985 |  | -6.42 | 2.4 | 0.007 | -1.68 | 4.1 | 0.680 |  | -0.02 | 0.03 | 0.55 | 0.04 | 0.06 | 0.46 |
|  |  | Q4 * TIC | -8.13 | 2.1 | 0.0001 | -2.99 | 3.5 | 0.390 |  | -9.07 | 2.5 | 0.0002 | -4.11 | 4.0 | 0.301 |  | -0.01 | 0.03 | 0.71 | 0.01 | 0.06 | 0.81 |
|  |  | P trend | -2.69 | 0.7 | <.0001 | -0.83 | 1.1 | 0.450 |  | -2.94 | 0.8 | 0.0001 | -1.16 | 1.3 | 0.360 |  | -0.004 | 0.01 | 0.62 | 0.004 | 0.02 | 0.83 |
| MID | Model 1 | Q1 | REF |  |  | REF |  |  |  | REF |  |  | REF |  |  |  | REF |  |  | REF |  |  |
|  |  | Q2 | 10.28 | 11.7 | 0.379 | -9.17 | 19.3 | 0.635 |  | 2.55 | 12.3 | 0.836 | -16.35 | 20.6 | 0.428 |  | 0.19 | 0.1 | 0.185 | 0.17 | 0.3 | 0.577 |
|  |  | Q3 | 7.89 | 13.2 | 0.549 | 17.25 | 21.5 | 0.422 |  | -5.58 | 13.8 | 0.685 | 8.53 | 22.8 | 0.708 |  | 0.25 | 0.2 | 0.123 | 0.29 | 0.3 | 0.403 |
|  |  | Q4 | NA | NA | NA | 4.34 | 26.6 | 0.871 |  | NA | NA | NA | -24.63 | 28.3 | 0.384 |  | NA | NA | NA | 0.81 | 0.4 | 0.061 |
|  |  | P trend | 4.93 | 6.4 | 0.443 | 4.00 | 7.9 | 0.620 |  | -1.93 | 6.7 | 0.774 | -3.97 | 8.4 | 0.636 |  | 0.14 | 0.1 | 0.093 | 0.23 | 0.1 | 0.072 |
|  | Model 2 | Q1 * TIC | REF |  |  | REF |  |  |  | REF |  |  | REF |  |  |  | REF |  |  | REF |  |  |
|  |  | Q2 * TIC | -1.19 | 1.8 | 0.502 | -3.04 | 3.4 | 0.376 |  | 0.42 | 2.1 | 0.839 | -2.27 | 3.9 | 0.563 |  | -0.05 | 0.02 | 0.041 | -0.06 | 0.1 | 0.319 |
|  |  | Q3 * TIC | -6.45 | 1.9 | 0.001 | -0.70 | 3.6 | 0.845 |  | -5.87 | 2.2 | 0.007 | 1.14 | 4.1 | 0.781 |  | -0.03 | 0.02 | 0.160 | -0.06 | 0.1 | 0.356 |
|  |  | Q4 * TIC | NA | NA | NA | -0.58 | 4.3 | 0.892 |  | NA | NA | NA | -3.12 | 4.9 | 0.525 |  | NA | NA | NA | 0.05 | 0.1 | 0.471 |
|  |  | P trend | -3.22 | 0.9 | 0.001 | 0.03 | 1.3 | 0.980 |  | -2.87 | 1.1 | 0.008 | -0.35 | 1.5 | 0.816 |  | -0.02 | 0.01 | 0.112 | 0.01 | 0.02 | 0.650 |

Deﬁnition of abbreviations: SE = standard error of mean; TIC = time in cohort; WBC = white blood count; NEU = neutrophilicgranulocyte; LYM = lymphocyte; MID = mid-range absolute count; REF = reference

^a^ Baseline WBC count and its differential were converted into categorical variables to assess the dose-response relationship with spirometry and its decline. Sex-specific range limits were used to categorize the study subjects into four groups with balanced sample sizes between groups for maximal statistical power and more reliable assessment of the dose-response relationship. Range of WBC count (*10^9^/L) levels for the four categories: for males, 1st quintile (2.3 – 5.1), 2nd quintile (5.1 – 6.0), 3rd quintile (6.0 – 7.1), 4th quintile (7.1 – 17.8); for females, 1st quintile (2.0 – 4.3), 2nd quintile (4.3 – 5.1), 3rd quintile (5.1-6.1), 4th quintile (6.1-14.5); 1st quintile was used as the reference group. Range of NEU (*10^9^/L) levels for the four categories: for males, 1st quintile (0.8 – 3.1), 2nd quintile (3.1 – 3.8), 3rd quintile (3.8 – 4.7), 4th quintile (4.7 – 13.1); for females, 1st quintile (0.5 – 2.6), 2nd quintile (2.6 – 3.3), 3rd quintile (3.3 – 4.0), 4th quintile (4.0 – 9.8); 1st quintile was used as the reference group. Range of LYM (*10^9^/L) levels for the four categories: for males, 1st quintile (0.6 – 1.6), 2nd quintile (1.6 – 1.9), 3rd quintile (1.9 – 2.3), 4th quintile (2.3 – 5.4); for females, 1st quintile (0.7 – 1.4), 2nd quintile (1.4 – 1.6), 3rd quintile (1.6 – 1.9), 4th quintile (1.9 – 8.4); 1st quintile was used as the reference group. Range of MID (*10^9^/L) levels for the four categories: for males, 1st quintile (0 – 0.2), 2nd quintile (0.2 – 0.3), 3rd quintile (0.3 – 2.3); for females, 1st quintile (0 – 0.1), 2nd quintile (0.1 – 0.2), 3rd quintile (0.2 – 0.3), 4th quintile (0.3 – 0.8); 1st quintile was used as the reference group. Males only had three groups for MID because of its discrete distribution. Model 1 assessed the associations of longitudinal lung function measurements as the outcome with baseline WBC and its differential with adjustment for age, smoking status (male only), packyears (male only), height, BMI, TIC, TWEI, and years of dust exposure. Model 2 assessed the association of baseline WBCs andlung function decline and used longitudinal lung function measurements as the outcome. In addition to all covariates adjusted in model 1, model 2 also included baseline spirometry and baseline WBC and its differential and interaction terms of TIC with TWEI, baseline spirometry, years of dust exposure, and baseline WBC and its differential. A negative β of the interaction term between TIC and baseline WBC in model 2 indicated that higher baseline WBC was associated with a more rapid decline of lung function. The quartiles of WBCs (Q1 as the reference group, Q2, Q3, Q4 coded as numerical 0, 1, 2, and 3) as categorical variables to assess the dose-response relationship with longitudinal lung function measurements and their declines firstly, then included as a continuous variable to test trend.

**Figure legend**

**Figure S1. Dose-response relationship between WBC count and its differential and FEV1 decline in workers by sex.** Baseline WBC count and its differential were converted into categorical variables based on sex-specific 25th percentile, median, and 75th percentile (see Table S7 for range limits). Linear mixed-effects model assessed the of quartile group status and FEV1 decline with Q1 as the reference. Q1, Q2, Q3, and Q4 group status was coded as numerical 0, 1, 2, and 3 in the trend test. The detailed modeling strategy was introduced in the footnote of Table S7. A significant dose-response relationship was identified for WBC, NEU, LYM, and MID in male workers only. A more rapid FEV1 decline was identified in male workers with higher levels of WBC or its differential. Only three groups for MID in males were available due to the discrete distribution of the MID data. A statistically significant test results (*) means P<0.05 compared to the Q1 group.


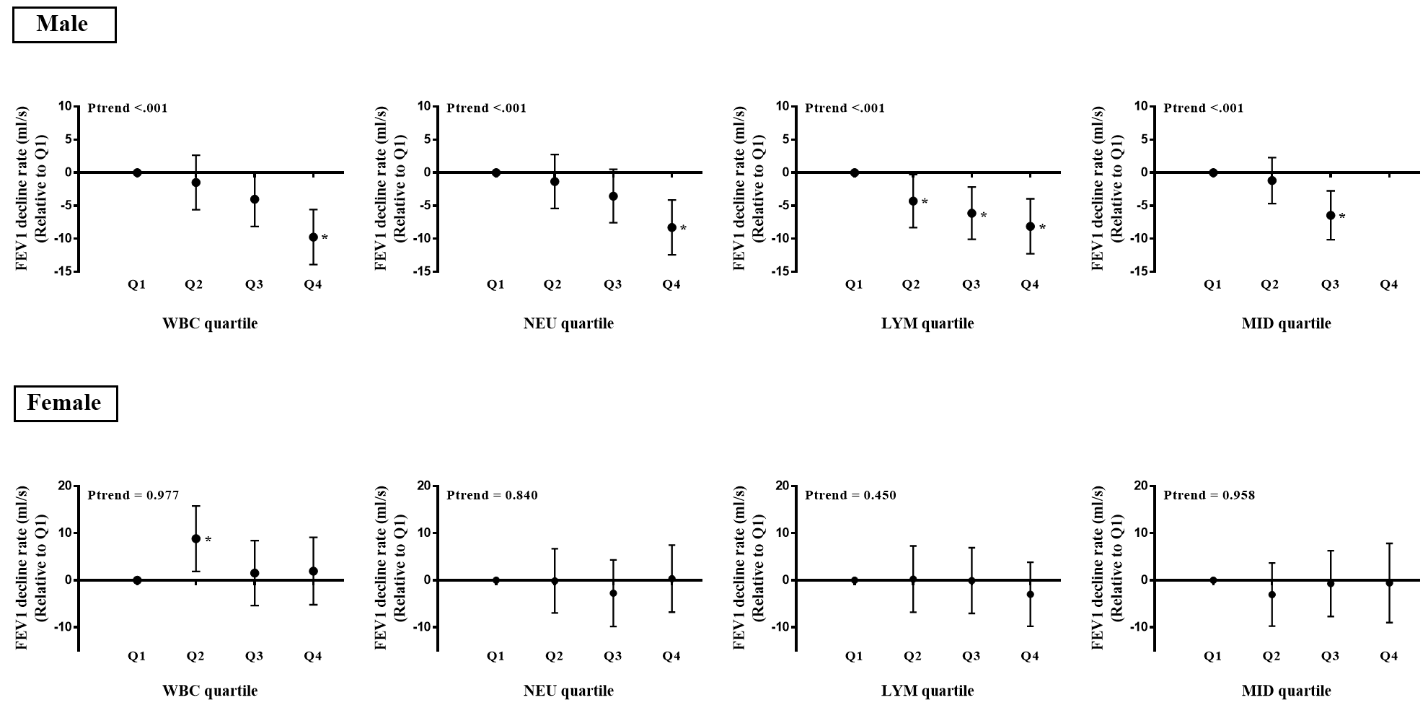

Supplement: Supplementary file 1 — Additional file 1: Table S1. Inhalable dust concentration (mg/m3) for different exposure category. Table S2. The association betwwen dust exposure and spirometry in female workers using linear mixeds-effects model (n = 1295). Table S3. The univariate analyses assessing the associations between time-weighted exposure intensity and characteristic variables as the outcomes. Table S4. The influence of inclusion of height and TIC interaction on the estimates and P values for TWEI and TIC interaction in male workers using linear mixed-effects model (n = 6100). Table S5. The influence of inclusion of height and TIC interaction on the estimates and P values for TWEI and TIC interaction in female workers using linear mixed-effects model (n = 1295). Table S6. The association between dust exposure and white blood cell count and its differential in female workers using linear mixed-effects model (n = 1295). Table S7. The association between categorized white blood cell count and its differential at baseline on longitudinal spirometry using linear mixed-effects model to assess the dose–response relationship. Figure S1. Dose–response relationship between WBC count and its differential and FEV1 decline in workers by sex. Baseline WBC count and its differential were converted into categorical variables based on sex-specific 25th percentile, median, and 75th percentile (see Table S7 for range limits). Linear mixed-effects model assessed the of quartile group status and FEV1 decline with Q1 as the reference. Q1, Q2, Q3, and Q4 group status was coded as numerical 0, 1, 2, and 3 in the trend test. The detailed modeling strategy was introduced in the footnote of Table S7. A significant dose–response relationship was identified for WBC, NEU, LYM, and MID in male workers only. A more rapid FEV1 decline was identified in male workers with higher levels of WBC or its differential. Only three groups for MID in males were available due to the discrete distribution of the MID data. A statistic [file 12931_2021_1849_MOESM1_ESM.docx]
